# Age and Sequence of Permanent Teeth Eruption in Lebanese Children

**DOI:** 10.1155/2020/9238679

**Published:** 2020-08-01

**Authors:** Nahla Nassif, Elia Sfeir

**Affiliations:** Department of Pediatric Dentistry, Lebanese University, Beirut, Lebanon

## Abstract

**Background:**

The timing of eruption of permanent teeth can be a precious help to the pediatric practitioner in identifying an appropriate treatment plan. Usually, it presents a more precise sign of an early or late growth of the child.

**Aims:**

It is to determine whether the timing of the permanent teeth eruption in Lebanese pediatric population can be considered as standard Lebanese values, since no other study was previously performed.

**Materials and Methods:**

A cross-sectional study is conducted, and the clinical emergence data are collected for a sample of 2317 Lebanese children aged between 5.5 years and 13 years (1129 girls and 1188 boys) from different socioeconomic groups in rural and urban communities spread over different regions in Lebanon. The study investigates dental emergence patterns.

**Results:**

Statistical analysis is performed with the SPSS Software. A confidence interval of 95% and significance level of 5% are adopted. The trend is for males to begin their permanent teeth eruption later than girls. Emergence of all permanent mandibular teeth is earlier than maxillary ones. Symmetry is found between the right and left teeth in the maxilla, as well as in the mandibula.

**Conclusion:**

In this study, the results indicate that premolars and second permanent molars erupt earlier in the Lebanese children compared to children of other countries reflected in several studies.

## 1. Introduction

Timing of teeth eruption is useful to the pedodontist and the orthodontist since it provides information from the standpoint of physiological growth and development. The skeletal age and dental age can be applied to assess the degree of physiological maturity of a growing child, as well [1].

The age determination of an individual by examination of teeth is an accepted method in the legal system.

In forensic dentistry, the age of subjects, with unknown birth record, can be calculated by using the emergence time of permanent teeth [2]. Legal systems, in general, accept the use of examination of teeth as an age determination mean [3].

Several studies have reported differences in permanent teeth eruption between ethnic groups [4–8]. Many physiological parameters are also found to have some effect on the eruption of permanent teeth: heredity, gender, puberty, nutrition, genetic, and socioeconomic and environmental factors [9–15].

So far, no eruption standards have been developed for Lebanese population. Consequently, there is a need to find out the emergence time and to establish the chart of sequence of eruption for Lebanese children. The purpose of the present study is to ascertain and to evaluate the timing of eruption of permanent teeth in Lebanese population and to compare with other groups.

## 2. Materials and Methods

This research was approved by the Ministry of Education, and an authorization was issued permitting access of schools to conduct this study. School lists were collected from the Center for Educational Research and Development (CERD). Private and public schools were chosen randomly in all Governorates in Lebanon to cover rural and urban areas. Before each visit, a consent form was forwarded to the students' parents by the school's administration.

A cross-sectional study was adopted with a sample of 2317 Lebanese school children (1129 females and 1188 males) aged between 5½ and 13 years. Children with systemic diseases, severe tooth size-arch length discrepancy, severe crowding, and those who have had early teeth extraction were not included in the study.

One operator performed the oral examination, by using a disposable dental checkup kit under daylight. The duration of the study extended between January and June 2003.

The developmental stage of the dentition was determined by grading each tooth according to its clinical eruption time. The grades were as follows:

1. Grade 0: tooth not visible in the mouth
2. Grade 1: tooth erupted with at least one cusp visible
3. Grade 2: tooth with at least two cusps or the 1/3 of the crown visible
4. Grade 3: tooth in occlusion or at the occlusal level

The children's age was calculated in months by subtracting the date of birth (included in students' record of the school) from the date of dental exam. Each tooth's dental eruption was noted only considering grade 1 and 2. Data collected were tabulated and subjected to statistical analysis using SPSS Software 11. Normal and *T* tests were employed.

A confidence interval of 95% and significance level of 5% were adopted.

## 3. Results

Table 1 shows the distribution of children sampled in Lebanon by gender (1129 females, 1188 males) and by age (from 5½ to 13 years). The smallest sample is 110 for the female's age group between 5 ½ and 6 years, while it is 131 for the male's of the same. The biggest female's sample is 184, for the age group between 9 and 10 years; as for the male's biggest sample, it is 190 for a different age group that is between 10 and 11 years.

Table 2 indicates the median age for eruption of permanent teeth in Lebanese children.

The median age for eruption of permanent teeth in Lebanese children is shown in Table 2. The mean age of eruption of permanent teeth is at 76.7 months for the first permanent upper right molar (#16), and it ends at 132.9 months for the second permanent upper right molar (#17).

There is a tendency for the lower teeth to erupt earlier than the upper ones. A difference of 7.3 months is noted between the central lower left incisor (#31) and the central upper left incisor (#21) which erupt, respectively, at 80.8 months and 88.1 months.

There are no statistically significant differences between the mean eruption age of the right and left permanent teeth. For tooth #17, the mean age of eruption is 132.9 months compared to 132.5 months for tooth #27. The mean age of eruption is at 123.0 months for tooth #15 and at 123.8 months for tooth #25.

Table 3 includes comparison in the eruption age of permanent teeth between females and males.

The permanent upper incisors and canines erupted earlier in females than in males. *P* values are 0,001, 0.027, 0.026, 0.046, 0.005,0, and 0.000, respectively, for the canine, lateral, and central incisors from the right to left side.

The eruption of the lower first right premolar (#44) is earlier in females (120.1 months) than in males (129 months) *P*=0.003. As for the lower second left premolar (#35), the eruption age for females is 123.1 months versus 127.2 months for males. The same trend applies for the canines and laterals on both right and left sides.

Table 4 shows the sequence of eruption in different countries. In Lebanon, the sequence of eruption of the permanent dentition is as follows: In the maxilla: 6-1-2-4-5-3-7 and in the mandibula: 6-1-2-4-3-5-7. In the maxilla, the sequence of eruption of the first molar, the central incisor, the lateral incisor, and the first premolar is the same in all the countries under comparison. The difference is observed in the second premolar which erupts before the canine in Lebanon and Iraq, contrary to other countries where it erupts later.

In the mandibula, the eruption sequence in Lebanese Children is similar to that of French and African children, where the first premolar erupts before the canine. In other countries under comparison, namely, Iraq, Pakistan, Ghana, and Northern Ireland, canine contrarily erupts before the first premolar.

## 4. Discussion

The study is based on children of different socioeconomic status, gathered randomly from villages and cities from all Lebanese Governorates.

According to the United Nation Program (UNP) and the Lebanese Ministry of Agriculture, 95% of Lebanese children are school students. The sample chosen from private and public schools is representative [23].

Tooth eruption is generally defined as the time when any part of the crown of the tooth has emerged through the gingival surface [8, 24, 25]. Authors generally have different definitions when it comes to “tooth eruption.” The reports utilized many terms for this purpose: the tooth is “just erupting” or “in any stage of eruption” or “fully erupted” or “present” or “broken through the gum” [26]. The process of tooth eruption is divided into five stages, one of which being mucosal penetration or emergence of any visible part of a tooth into the oral cavity [24]. There is a difference between a tooth in occlusion and a tooth that has at least one cusp visible in the mouth. Sometimes, 10 to 12 months are required in order for a tooth to get from the gum to the occlusion [27].

In this study, only stages 1 and 2 are taken into consideration. They represent the mean period between all stages of tooth eruption.

The manner of recording age is carefully determined in months, which is more accurate than calculating in years, or calculated to the nearest month and later recorded into 4 month groups [12, 25, 28].

Our results agree with those of different studies, regarding similar chronology of eruption of contralateral teeth, earlier eruption of mandibular teeth compared with maxillary ones, and the differences between girls and boys [5, 12, 24, 25, 28].

The sequence of eruption of the upper permanent teeth in Lebanese children (6-1-2- 4- 5- 3- 7) is different from that in Nepali [16], Pakistani [18], Australian [19], African [20] and Caucasian [21], but it is identical to the values of Iraqi children [17].

In the mandibula, the sequence of eruption of permanent teeth is different from many populations, but similar to the results found by Tisserand–Perrier (1958): The second premolar erupt before the first premolar and before the canine [22] (Table 4).

A descriptive comparison in eruption chronology is performed with other countries [17, 20, 28]. The eruption age of the Lebanese children is delayed when compared to the Canadian and Finnish ones. This delay could reach 8 months in girls in the lower central incisor.

The first premolars in Lebanese children erupt earlier than in Finish and Caucasian children [20, 28]. On the other hand, eruption is delayed when compared to Ghanaian and Tanzanian children [20, 29] (Table 5).

Differences between the Lebanese and Caucasian children, as well as some similarities between Lebanese and Ghanaian Children, should be considered valid and cannot be ignored for the following facts:

1. The numbers are sufficient for a statistical study. Indeed, the number of the 2nd upper right premolars is 152, 177 for the 2nd upper left premolars, 161 for the 2nd lower left premolars, and 146 for the lower right premolars. The same goes for the 2nd upper and lower left and right molars that ranged from 108 to 187 (Table 2).
2. WHO recommendations are strictly followed for the oral exam [30].
3. Even though most children have their 2nd molars already in occlusion at the age of 12, this study included children of age 13, thus widening the scope.

One of the explanations of the differences shown in this study is that the Lebanese population might not be purely Caucasian. It is known to be a mix of different ethnicities. The differences found can also result from numerous factors such as the environment, type of food, dental caries, lifestyle, general health, and genetic variations. The premature loss of deciduous teeth due to caries can also accelerate the eruption of their replacements.

## 5. Conclusions

Usually, in clinical and academic situations, American and European standards are used. However, the research results are specific to the Lebanese children.

Eruption Sequences presented in this study are meant to serve as general guidelines for the Lebanese Population. The determination of the dental age of a child is always complex, although it might seem to be relatively simple. Lebanese children have advanced eruption of the permanent premolars and the second molars compared to Asian, European, and Iraqi children. Comparable values are also observed in African children who usually have an earlier eruption.

## Figures and Tables

**Table 1:** Distribution of the Lebanese sample (age, females, males, and total).

Lebanon			
Age in years	Females	Males	Total
(5, 5–6)	110	131	241
(6–7)	166	184	350
(7–8)	164	170	334
(8–9)	176	176	352
(9–10)	184	170	354
(10–11)	179	190	369
(11–13)	140	167	317
Total	1129	1188	2317

**Table 2:** The age of eruption of the permanent teeth of Lebanese children (age in months).

Tooth	Lebanon		
	Total *n*	Mean age (months)	Standard deviation
17	125	132.9	10.03
16	214	76.7	9.69
15	152	123.0	12.62
14	303	119.9	13.95
13	200	128.8	10.82
12	301	100.8	13.00
11	265	88.0	9.92
21	255	88.1	8.84
22	293	100.5	12.41
23	181	128.2	10.74
24	291	119.8	13.53
25	177	123.8	12.76
26	205	77.0	8.74
27	108	132.5	11.08
47	178	131.4	11.02
46	161	78.7	10.16
45	146	126.4	12.12
44	254	122.5	12.69
43	269	123.3	11.82
42	376	93.5	10.16
41	260	80.8	7.78
31	261	80.8	8.04
32	352	94.0	9.96
33	275	123.3	11.26
34	303	122.2	12.79
35	161	125.1	12.41
36	181	77.7	9.04
37	187	130.9	11.16

**Table 3:** Comparison of the age of eruption of the permanent teeth between females and males in Lebanese population (age in months).

Lebanon							
Tooth	Females			Males			*P* value
	Total *n*	Mean age (months)	Standard deviation	Total *n*	Mean age (months)	Standard deviation	
17	61	131.4	10.7	64	134.3	9.3	0.111
16	92	77.9	11.9	122	75.8	7.5	0.139
15	66	121.7	12.6	86	124.0	12.6	0.256
14	146	118.5	13.4	157	121.3	14.4	0.084
13	100	126.3	11.3	100	131.2	9.8	0.001
12	154	99.2	13.1	147	102.5	12.7	0.027
11	136	86.6	10.2	129	89.3	9.5	0.026
21	135	87.0	8.5	120	89.3	9.1	0.046
22	150	98.6	11.9	143	102.6	12.7	0.005
23	89	124.7	11.1	92	131.7	9.2	0.000
24	145	118.8	12.5	146	120.8	14.5	0.193
25	76	121.9	13.5	101	125.3	12.0	0.087
26	85	76.5	8.9	120	77.4	8.7	0.473
27	60	133.0	10.6	48	131.9	11.7	0.618
47	91	130.9	10.3	87	131.9	11.8	0.554
46	67	78.9	11.3	94	78.5	9.3	0.804
45	75	125.5	11.2	71	127.3	13.0	0.353
44	125	120.1	12.6	129	124.7	12.5	0.003
43	134	120.3	10.7	135	126.3	12.2	0.000
42	212	91.7	9.8	164	95.7	10.2	0.000
41	118	80.6	8.1	142	81.0	7.6	0.694
31	117	80.7	8.5	144	80.9	7.7	0.882
32	195	92.4	9.5	157	96.1	10.2	0.001
33	134	120.1	10.2	141	126.4	11.4	0.000
34	151	121.1	12.2	152	123.3	13.3	0.150
35	84	123.1	11.1	77	127.2	13.4	0.033
36	79	77.4	9.1	102	77.9	9.1	0.742
37	88	129.5	10.9	99	132.1	11.3	0.114

**Table 4:** The sequence of eruption of the permanent dentition in different countries.

Country	Maxilla	Mandibula
Lebanon (present study)	6 1 2 4 5 3 7	6 1 2 4 3 5 7
Nepal [16]	6 1 2 4 3 5 7	6 1 2 3 4 5 7
Iraq [17]	6 1 2 4 5 3 7	6 1 2 3 4 5 7
Pakistan [18]	6 1 2 4 3 5 7	6 1 2 3 4 5 7
Australia [19]	6 1 2 4 3 5 7	6 1 2 3 4 5 7
Ghana [20]	6 1 2 4 3 5 7	6 1 2 3 4 5 7
Northern Ireland [21]	6 1 2 4 3 5 7	1 6 2 3 4 5 7
France [22]	6 1 2 4 3 5 7	6 1 2 4 3 5 7

**Table 5:** The age of eruption of the permanent teeth in different countries (age in years).

Tooth	Iraq [17]				Ghana [20]				Canada [20]				The United States [20]			
	Maxilla		Mandibula		Maxilla		Mandibula		Maxilla		Mandibula		Maxilla		Mandibula	
	Boy	Girl	Boy	Girl	Boy	Girl	Boy	Girl	Boy	Girl	Boy	Girl	Boy	Girl	Boy	Girl
1	7.4	7.4	6.2	6.2	6.3	6.0	5.3	5.1	7.2	6.5	6.1	5.5	7.4	7.2	6.54	6.25
2	8.7	8.3	7.6	7.5	7.5	7.3	6.1	6.4	8.3	7.95	7.3	6.9	8.6	8.2	7.70	7.34
3	11.5	10.9	10.6	10.0	10.4	9.5	10.0	8.9	11.4	10.6	10.7	9.6	11.69	10.98	10.79	9.86
4	10.0	10.0	10.6	10.2	9.5	9.0	9.8	9.2	10.5	10.05	10.8	10.2	10.40	10.03	10.82	10.18
5	10.9	10.8	11.4	11.0	10.5	10.0	10.6	10.3	11.3	11.2	11.7	11.1	11.8	10.88	11.47	10.89
6	6.1	6.0	5.7	5.7	5.0	5.0	4.9	4.5	6.1	5.4	6.2	5.8	6.40	6.22	6.21	5.94
7	12.2	11.8	11.8	11.4	10.9	10.9	10.8	10.5	12.3	12.3	12.0	11.5	12.68	12.27	12.12	11.66

## Data Availability

Data can be made available upon request.
